# “Noah’s Ark” Project: Interim Results and Outlook for Classic Collection Development

**Published:** 2018

**Authors:** M. V. Kalyakin, A. P. Seregin, A. E. Solovchenko, P. A. Kamenski, V. A. Sadovnichiy

**Affiliations:** M.V. Lomonosov Moscow State University, Leninskie Gory, 1, Moscow, 119991, Russia

**Keywords:** Animals, biobank, depository, microorganisms, plants

## Abstract

The “Noah’s Ark” project, afoot at M.V. Lomonosov Moscow State University since 2015 and aimed at studying biodiversity, is the largest ongoing Russian project in life sciences. During its implementation, several hundred new species have been described; a comprehensive genetic and biochemical characterization of these species, as well as that of the pre-existing specimens in Moscow University’s collections, has been performed. A consolidated IT system intended to house the knowledge generated by the project has been developed. Here, we summarize the investigations around the Moscow University classical biocollections which have taken place within the framework of the project and discuss future promise and the outlook for these collections.

## INTRODUCTION

There is little doubt that, in the near future, our existence will be largely governed by so-called “big data”: huge arrays of information whose effective use is already revolutionizing many aspects of human life. In the field of life sciences, the term “big data” is traditionally associated with genomic information; i.e., the results of sequencing of many genomes. However, genomic data is just one example of real “big data” generated by life sciences; namely, by profound studies of biological collections. A biological collection is defined as an organized repository of biological specimens of any kind – from dried plants to living human cells, and even sequenced genomes.

It is becoming increasingly clear that the potential of biological collections is significantly higher than has been commonly accepted. However, to harness this potential, one must treat biological collections as sources of “big data” – vast amounts of information about living systems. Combining this information with the modern techniques of life sciences would allow us to obtain invaluable insights into the origin and evolution of life on Earth. This is expected to arise from comparative studies of numerous biological samples. The resulting knowledge could be implemented in practice for the preservation of the biodiversity of our planet.

This was the guiding principle behind Noah’s Ark, a project dedicated to the conservation, investigation, and profitable use of biological diversity. The most important prerequisite for successful implementation of the project was the creation of a unified virtual space for biocollections with the potential to harvest diverse data on a virtually unlimited number of biological samples. Such a space has already been created, so far on the scale of Moscow State University, but there are plans to make it nationwide. It is already obvious that a global approach to the study of biodiversity significantly increases the quality of scientific results, allowing us to identify more general, and more complex, patterns in the organization of life on our planet.

Here, we review the interim results of Noah’s Ark project for classical biological (animal, plant, and microbiological) collections.

## ANIMALS

The purpose of a biobank is to accumulate collections that adequately reflect the multidimensional structure of biodiversity (BD), making it possible to explore its various manifestations. An analysis of the scientific status of zoological collections was carried out [1], and it was shown that the collections perform the function of a research sample in BD studies. Their main characteristics are their representativeness, which is further detailed by their informational value, reliability, systemic character, scope, structure, etc.

The studies in the “Animals” section are aimed at analyzing key aspects of BD on the basis of an integrated approach combining phylogenomic and phylomorphological analyses of data resulted from electron microscopic and 3D reconstructions data.

The macrotaxonomic analysis of the main Animalia groups included taxa ranging from order to phylum. One fundamentally new finding is the reliable substantiation of the monophiletic status of the Lophophorata clade including Phoronida, Brachiopoda, and Bryozoa: it is supported by the architecture of the coelomic system and innervation of lophophore tentacles [2-9]. This conclusion is of crucial importance for elucidating the structure of the phylogeny of animals at the level of the Metazoan basal radiation. The study of phylogenetic relationships in the Ophiuroidea class was also one of the breakthroughs achieved. It is divided into the Euryophiurida and Ophintegrida superorders, and four new orders and 11 families have been recognized [10]. Essentially new results were obtained for the classification of the Nudibranchia (Mollusca) order, in which three new families were described [11]. The importance of pedomorphosis in the formation of new taxa of high rank and the need to study the diversity of ontogenetic patterns for their identification has been demonstrated within the framework of the ontogenetic systematics concept [12-15]. The molecular phylogenetic analysis demonstrated the monophily of eight genera of the Acrothoracica (Copepoda) superorder [16]. The analysis of the generic composition revealed 24 new taxa of this rank in the Gastropoda, Maxillopoda, and Mammalia classes [11, 17-22]. It is obvious that a sole phylogenomic approach to the analysis of the structure of macrotaxonomic diversity is insufficient: it should be supplemented by a study of morphological diversity at the level of ontogenetic patterns. This is consistent with the most recent ideas about the evo-devo concept according to which the historical development of multicellular organisms is mainly an evolution of their ontogeny; in macrotaxonomic studies, these ideas are developed through the concept of ontogenetic systematics.

The microtaxonomic analysis of species and subspecies was carried out on the basis of the concept of integrative taxonomy: species were identified using genetic material, then the results were clarified using morphological and epiphenotypic (including acoustic) characters.

New species and subspecies of animals were identified (their number is indicated in brackets) in Cercozoa (4) [23, 24], Cnidaria (1) [25], Kamptozoa (6) [21, 26], Phoronida (5) [3, 27], Nematoda (13) [28, 29], Annelida (9) [30-33], Chaetognatha (1) [34], Mollusca (27) [11, 17, 18, 35-41]; Maxillopoda (23) [42-46], Arachnida (2) [47, 48], Insecta (48) [49-60], Osteichthyes (7) [61-63], Amphibia (16) [64-67], Reptilia (14) [68], Aves (4) [69], and Mammalia (4) [22, 70]. The possibility of a reliable identification of the related species and subspecies of a number of Asian Insecta, Amphibia, Reptilia, Aves, and Mammalia by acoustic parameters was demonstrated for the first time [71-76]. A method has been developed for determining the genetic identity of jellyfish and polyps in the laboratory lines of some Cercozoa, which makes it possible to adequately assess the diversity of these species [77]. The specific and subspecific levels of taxonomic differentiation of the Asterocheridae and Ascothoracida groups have been marked [43, 45]: correct differentiation of these levels is one of the key challenges of microsystematics.

The combination of genomic phylogeography and genetic barcoding provided new data on the structure of species diversity in a number of animal groups. In the Nothybidae (Insecta) family [78], several economically important species of Salmonidae and Cyprinidae (Osteichthyes) [79-84] and species-level taxa from 10 families of terrestrial vertebrates in Eurasia have been studied in this respect [64, 85-95]. The high efficiency of *COI *gene analysis in revealing the diversity of phylogenetic lineages in a number of *Amphibia *genera has been demonstrated [96].

A preliminary study of the molecular genetic and morphological diversity of representatives of the Megophryidae, Dicroglossidae, Microhylidae, Rhacophoridae (Amphibia), and Gekkonidae (Reptilia) families revealed a high level of “hidden” species diversity, which requires a more detailed study. Comparative analysis of the geographic variability of some model Palaearctic bird species (Aegithalidae, Sylviidae, Corvidae families etc) indicates a group-specific nature of their intraspecific differentiation. [97]. Active reticular microevolution has been shown to occur in the genus *Darevskia *(Reptilia) [98]. Revealing the complex of sympatric forms of the genus *Salvelinus *(Osteichthies) suggests their sympatric speciation [81, 82]. It was found that local populations of *Hypomesus olidus *and *Salvelinus malma* (Osteichthies) are being formed as independent units in isolation on the Commander Islands [99, 100]. On the basis of a comprehensive analysis of fish from several families, weak agreement of divergence of population and species units in morphogenetic characteristics and the presence of a large number of cryptic species have been demonstrated; the species diversity of the studied groups of animals is significantly underestimated. Therefore, the key task is to translate the “hidden” diversity into an “obvious” one through collection and storage, including those of new forms of collection material, and the development of new methods for analyzing species differentiation.

Within the results of a study of the meronomic diversity of animals, the most impressive is the demonstration that the miniaturization of insects of the Coleoptera (fam. Ptiliidae), Psocoptera (fam. Liposcelididae), and Thysanoptera orders, which are comparable in size to unicellulars (about 1 mm), has almost no effect on the anatomy of the most important organs of the head section [101, 102]. The result is of fundamental importance for understanding the mechanisms that ensure conservation of the structure of multicellular animals. A new type of oogenesis, autoheterosynthesis, has been described in Phoronida [103], which expands our comprehension of the diversity of ontogenetic patterns. A mechanism for the emission of sound signals has been, for the first time, discovered in representatives of a number of Orthopteran and Homopteran families, suggesting repeated formation of a similar stridulation signal during their evolution [71]. The results of the analysis of vibration and sound signals in the species of a number of Orthoptera and Homoptera families [71-73, 77] confirm the hypothesis that they serve as an effective reproductive barrier. Cranial differences in isolated populations of Arctic fox *Vulpes lagopus *on the Commander Islands were shown to result from selection, rather than genetic drift [104].

In the studies of the biochorological section, the faunistic complexes of invertebrates and vertebrates of the seas of the Arctic Basin, the Russian Far East, the North Atlantic, the Australasian tropical seas, and the Red Sea were examined. An analysis of the diversity of representatives of five Nematoda groups of hydrothermal sites of the Mid-Atlantic Ridge at depths of 1,200–1,500 m [105, 106] was conducted. In terms of taxonomic composition and biological characteristics, hydrothermal nematodes differ from deep-water bathyal and abyssal nematodes, but they are similar to shelf and sublittoral species and communities. It has been shown that the faunistic diversity of marine benthic heterotrophic representatives of Flagellata in the World Ocean is more consistent with the predictions of the “cosmopolitanism” model rather than “moderate endemism” [107]. It is shown that the Harpacticoida fauna at low latitudes is much richer and has a significantly higher degree of endemism compared to the fauna of high latitudes; the populations of shallow (up to 50 m) and deeper zones differ in species composition. A significant difference between the harpacticoid faunas of the Eastern and Western parts of the Arctic seas has been revealed [108]. The composition of the Laptev Sea macrobenthos and its diversity revealed the presence of a general bathymetric trend: one set of factors affects both the composition and functioning of benthic communities [109]. It has been established that differences in the composition of the Cladocera freshwater fauna of the Arctic and Subarctic zones are determined primarily by modern climatic factors, which makes it possible to use these faunistic complexes as bioindicators [110]. Large-scale studies of the invertebrate species composition of the Arctic and Far Eastern seas have been carried out, and new data on representatives of Ciliophora and Kamptozoa have been obtained [109, 111-113]. A relationship between genetic, morphological, and taxonomic diversity in the four Annelida families from the fauna of the northern seas has been revealed [109]. The species composition of the Cladocera of the freshwater lakes and shallow seas of Asia was clarified [114, 115]; it has been found that the Cladocera fauna of the coastal waters of Borneo is significantly poorer than the mainland one [116]. Four types of communities of shell amoebas (Testacea) were identified in the basin of the Belaya River [117].

The development of an integrated approach to long-term monitoring of the spatial dynamics of species and faunistic diversity based on the regular collection and analysis of monitoring collections in the focal regions of northern Eurasia is of fundamental importance [118]. It allows for the identification of regions with potentially increased vulnerability for biodiversity and proposing measures for its conservation.

The study of the ecological aspect of BD mainly centers on the analysis of the spatial dynamics of the energetics of birds inhabiting different environments. A significant specificity of the energy of Old World tropical birds was confirmed. In particular, it was demonstrated that the absence of a phylogenetic signal in basal metabolism is independent of body weight [119].

## PLANTS

Reconstruction of the origin, spread, and kinship of various groups of plants in the project is achieved through a wide use of molecular methods in classical science.

In the Fabaceae family, the results of a long-term molecular-genetic and morphological analysis of wild bird-foots made it possible to reconstruct not only the evolution of the *Lotus *genus, but also the key points of the historical biogeography of the group [120]. The independence of families close to the bird-foots *Hammatolobium*, *Tripodion, *and *Cytisopsis *was also demonstrated [121]. The history of the *Lagochilus *genus from the Lamiaceae family was also reconstructed [122]. It was shown that the diversification of this Central Asian genus is directly related to recent geological history and subsequent climatic shifts. In the Apiaceae family, the scope of intrageneric divisions in the *Prangos *genus has been revised based on a DNA analysis and a new *Koelzella *subgenus has been established [123]. In turn, the “forgotten” Afghan endemic *Prangos akymatodes *[124] was restored as a separate species within. In addition, in order to ensure monophyletism, the monotypic *Alococarpum *genus was transferred to the *Prangos *genus [125].

The integrative molecular-morphological approach allows not only to establish the origin and relationship of taxa, but also to restore the most probable course of evolution of individual features. For example, the presence of single-seeded fruits from a common ancestor of the Caryophyllales order, numbering 12,000 species, has been established [126]. A detailed description of the seeds of the polyphyletic *Mollugo *genus was provided, which made it possible to draw important conclusions for the classification and taxonomy of groups [127]. A consistency of seeds structure features with the latest molecular data has also been demonstrated for Caucasian species of the *Minuartia *genus [128].

Molecular phylogenetic analysis was used to demonstrate the need to revise many groups of moss. The most illustrative example is the polyphilia of the Ditrichaceae family: a detailed analysis convincingly demonstrated that characteristics that were considered to be taxonomically significant appeared independently in different groups [129]. Based on this relationship, a new order and three new moss families have been described [130]. Further revision of individual groups of mosses led to a significant revision of relations in the Grimmiales order [131].

Attempts to solve the particular problem of describing a new species of *Bryoerythrophyllum duellii*, using molecular data not only for this genus, but also for its immediate relatives, made it possible to completely revise the scope of the *Bryoerythrophyllum *genus [132].

An in-depth study of the genomes of flowering plants and mosses has been performed. The full plastomas of three types of *Dryopteris*, *Adianthum hispidulum *[133], *Seseli montanum *[134], and some others, has been deciphered and annotated. The structure of the intergenic spacer IGS1 of the ribosomal operon in moss of the *Schistidium *genus was studied in detail [135].

An example of a monographic study that combines both the classical morphological approach and the latest molecular methods is the processing of herbarium specimens of wild onions from the *Allium saxatile *group [136]. Of the 15 species, five were new to science. Geographic isolation was the main cause of previously underestimated speciation: researchers were able to describe new species from Romania, Bulgaria, Russia, Kazakhstan, and China. Later, another type of onion from Uzbekistan [137] and another one from Turkey [138] were described.

A monographic revision of the recently described *Paramollugo *(Molluginaceae) genus, which, as expected, consists of only three species, has doubled the number of known species [139]. Two new species are described for Madagascar (*Paramollugo simulans *and *P. elliotii*), and another “forgotten” species was found in collections from New Caledonia.

Another successful example of monographic processing is the revision of the African *Corbichonia *(Lophiocarpaceae) genus, which included only two species [140]. A third species, *C. exellii*, which is spread over several countries of Southern Africa at once, has been discovered and certified.

The results of the revision of the *Rhabdosciadium *genus from the Apiaceae family have been published, which includes seven species distributed in the mountainous areas of Turkey and Iran. It was possible to analyze the DNA of all members of the genus, including several narrow-local endemics. The monophyletism of this genus has been demonstrated, and a new species, *R. anatolyi, *common in Turkish Kurdistan [141], has been described. A new species of endemic umbellate from Laos has been found: *Xyloselinum laoticum* [142].

The traditional study of the systemic structure and taxonomy of the Chenopodiaceae family has been extended. A new species, *Dysphania geoffreyi*, has been described for areas hard-to-reach for European researchers, such as Lhasa and Bhutan [143]. Subsequently, *Atriplex congolensis *orach from the Democratic Republic of the Congo [144] and the *Arthrocnemum franzii *saltwater from the Cape Verde Islands [145] have been described.

According to the results of an extensive revision of the genus *Atraphaxis*, several new taxa of the Polygonaceae family have emerged: *Atraphaxis kamelinii *species from Mongloia [146], *Bactria *genus with *B. lazkovii*species from Kygryzstan [147], and the *Persepolium *genus [148].

For reasons of nomenclature, *Calciphilopteris wallichii, *a new species of fern from the Philippines, had to be re-described [149]. We would also like to note the description of a new species of moss *Schistidium relictum *[150], widespread in Canada and Russia.

Refining of our knowledge of the geographical distribution of organisms follows two paths: studying existing collections that had not previously been described accurately and field studies. As a result of this work, a whole layer of new data has been acquired, which is referred to as “floristic finds” [151].

One of the most remarkable discoveries is that of *Scapania aspera *earwort. It was possible to find in nature, correctly recognize, and subsequently perform a DNA analysis of the plant found on the Anabarsky plateau, 3,000 km from the nearest known habitats of this earwort in Europe [152].

Floristic finds are merely at the top of a huge reservoir of information that accumulates as a result of a floristic survey of any territory. The results of such work are reflected in the “Floras” and checklists. For example, the results of studying the flora of Sevastopol were summarized. It has been shown that the western extremity of Mountainous Crimea is one of the most floristically rich corners of Russia, with 1,859 species of vascular plants recorded in an area of about 600 km2 [153].

Important results were obtained in the field of palelinology during the course of the project. Mass pollination of plants can be considered not only as a biological process, but also as a special natural phenomenon that can be studied from the standpoint of botany, meteorology, paleogeography, and allergology.

A group of palynologists analyzed long-term data on birch pollination in the Moscow region and identified the main meteorological factors affecting the concentration of pollen during its season [154]. A comparative study of urban and suburban pollen spectra showed that pollen monitoring station data collected in large cities can be extrapolated to the surrounding countryside [155].

Traditional studies of the morphology and anatomy of pollen and spores were extended: heterosulcate pollen grains of the swamp forget-me-not *Myosotis scorpioides *and their development were described in detail [156], as well as the structure of sphagnum moss spores at different stages of germination [157].

Herbarium samples are an important and easily accessible source for the selection of DNA samples, but DNA molecules are gradually destroyed during storage. Therefore, the method developed for extracting DNA from old herbarium specimens deserves special attention [158].

Translating these collections into electronic form or, in other words, virtualization of the collection space was conceived as a mainstream development in the study of plants. The large project on the digitization of the Herbarium of Moscow State University was used as the basis for this work [159].

## MICROORGANISMS AND FUNGI

Within the “Microorganisms and fungi” section, a comprehensive depository of bacteria, fungi, fungi-like organisms (myxomycetes and oomycetes), and algae has been created. A unique array of information about the microorganisms has been compiled, along with extensive collections important for scientific research as well as for practice. The uniqueness of the biomaterial collections and knowledge accumulated within the framework of the Project rests in its complexity and the scope of the biodiversity captured in the collections and in the diversity of the habitats screened. Microbial communities of soils of different natural zones, urbanized biotopes, habitats with extreme conditions have been characterized [160]. An important aspect was the study of the soil microbial communities of Antarctica [161-165]. The dominant fungi in moss-covered Antarctic soil were those from the genera *Phoma, Thelebolus, Penicillium, Rhodotorula*, in “cobblestone pavement”, *Cadophora, Cladosporium, Cladophialophora*, in aquatic habitats, *Antarctomyces, Hyphozyma, Goffeauzyma, Phoma, Thelebolus, and Geotrichum*.

Another group of fungi, macromycetes, was the focus of research on diversity, ecology, and potential practical use. This group encompasses major decomposers closing the nutrient loop in ecosystems. Rare species were found [166], and a number of new species of macromycete fungi have been described [167-169]. The study of urbanized ecosystems was equally important, as was the inventory and quantification of potentially pathogenic fungal species in the soil [170, 171] and plant pollen [172]. Micromycete complexes enriched with species potentially hazardous to health and causing bio-damage are forming in urban soils [171]. On the other hand, parks and botanical gardens created in cities are refugia for rare and interesting species of mushrooms and myxomycetes. The previously unexplored and poorly described features of yeasts from diverse soil types and biocenoses have been studied: soils in the temperate zone of Russia [173], soils under the thickets of invasive plants (such as *Heracleum sosnowskyi*) [174-176], soils under the vineyards of Dagestan [177, 178], and plantations in South Vietnam [179]. Overall, the soils turned out to be a natural reservoir of yeast biodiversity.

Fungi and fungi-like organisms (myxomycetes and oomycetes) are extremely important both for the functioning of ecosystems and for human practice. Their ubiquity obviates the need for collections encompassing many different regions for studying these organisms. It is important to cover both reference habitats in natural reserves and anthropogenically impacted areas. The Project made it possible to carry out unprecedentedly broad studies of the diversity of soil microscopic fungi and myxomycetes of nature reserves (the Central Forest Biosphere Reserve, the Kaluga Zaseki Reserve, the Volga-Akhtubinskaya Floodplain Natural Park) [180]. Extensive data on the diversity and distribution of microscopic fungi in the protected forests of Vietnam were collected both for cultivated and uncultivable species, as well for myxomycetes [181].

The collections created within the framework of the Project became a unique database for studies of practically important microorganisms. Strains–producers of broad-spectrum antibiotics from the peptaibol family [182], the anticancer metabolite Brefeldin-A, as well as potential steroid producers, have been identified [183]. The study of phytopathogenic fungi in both natural habitats, which are reservoirs of phytopathogens, and in agrocenoses is of great interest and practical importance [184]. Extensive collections were created and used as the basis for population studies of the most dangerous potato pathogens, *Phytophthora infestans *[185] and *Alternaria *[186]. Their population features and mechanisms of fungicide resistance have been identified [187, 188]. Among the huge diversity of microorganisms inhabiting different soil horizons, yeast fungi deserve special attention as one of the most biotechnologically significant groups of microorganisms [189].

Identification of the most resilient microorganisms in the extreme natural habitats of the Earth is among the most important tasks of microbiology, largely unsolvable without the study of ancient rock sediments. Gamma-ray resistance of the microbial communities from permafrost sedimentary rocks of the Arctic was studied by exposure to gamma radiation (100 kGy) in low temperature (–50 °C) and low pressure (1 mmHg) conditions. These results can be considered as terrestrial models of the conditions encountered by microorganisms in the regolith habitats on Mars. Microbial communities of permafrost showed high resistance to the simulated harsh extraterrestrial conditions, retaining ample cultured, metabolically active prokaryotes [190]. The results obtained indicate the possibility of long-term cryopreservation of viable microorganisms in the Martian regolith. Taking into account the intensity of radiation on the surface of Mars, our data suggest the possibility of conservation of hypothetical Mars ecosystems in the regolith layer (e.g. protected from UV rays) for at least 1.3–2 mln years. At a depth of 2 m (the estimated sampling depth of the ExoMars 2020 mission), the viability expectance is at least 3.3 mln years, and at a depth of 5 m–at least 20 mln years. Of particular interest are microscopic fungi naturally adapted to extreme salinities and alkalinities. Therefore, a collection of isolates from the White Sea marshy habitats [191] and soda solonchaks [192] was a focus of the project, generating a plethora of physiological and biochemical studies. Important stress tolerance mechanisms associated with the structure of membranes were deciphered using these collections [193].

## CONCLUSIONS AND OUTLOOK

The abovementioned studies convincingly demonstrate the scientific potential in approaching biocollection studies globally. Such an approach presumes, for example,, comprehensive analyses of large numbers of samples regardless of their (zoological, botanical or microbiological) nature. Moreover, the depth of the insight from comparative studies seems to linearly or even exponentially depend on the number of specimens involved. Therefore, the number of biological specimens available should be maximized by every means for further progress in comprehensive biological studies. We believe that the natural way to achieve this is to embrace as many biological collections as possible in a consolidated data environment. The prototype of such an environment has already been created in the form of the IT-system of the Noah’s Ark project (https://depo.msu.ru/). As of March 2018, the IT -system contained information on more than a million biological specimens. Making this system nationwide will provide a powerful impetus to the development of life sciences in Russia and to the translation of the fundamental research results into practice. We envision the extension of the IT-system of the Noah’s Ark project towards the main directions of its development.

The success of the Noah’s Ark project is largely due to its interdisciplinary character. Implementation of the project was the main driver behind fulfilling the long-held dream of classical biocollection owners at MSU–creating genetic and biochemical service labs focused on maintenance of these collections. Of course, specimens of these collections had been studied before, but that activity was of secondary importance to other laboratories in the project, naturally affecting their efficiency. At present, any specimen newly deposited in a MSU collection is subjected to thorough genetic and, in many cases, biochemical characterization. It also became possible to analyze DNA extracted from museum samples. Of special importance is the possibility of comprehensive microscopic studies. It is clear that such an integrated approach will be much more insightful. There is also little doubt that the synthesis of the classical and modern research methods implemented in the project must be fully endorsed and further developed in other research areas.

Collectively, we envision (i) the extension of the project IT nationwide and, later, internationally as well as (ii) the elaboration of new advanced genetic, biochemical, and physico-chemical tools that would be used to analyze specimens from biocollections as the main avenues for further development of the Noah’s Ark project.
